# Impact of the Renin-Angiotensin System on the Pathogeny and Pharmacotherapeutics of Neurodegenerative Diseases

**DOI:** 10.3390/biom12101429

**Published:** 2022-10-06

**Authors:** Walther Bild, Alexandru Vasincu, Răzvan-Nicolae Rusu, Daniela-Carmen Ababei, Aurelian Bogdan Stana, Gabriela Dumitrița Stanciu, Bogdan Savu, Veronica Bild

**Affiliations:** 1 Department of Physiology, “Grigore T. Popa” University of Medicine and Pharmacy, 700115 Iasi, Romania; 2 Center of Biomedical Research of the Romanian Academy, 700506 Iasi, Romania; 3 Department of Pharmacodynamics and Clinical Pharmacy, “Grigore T. Popa” University of Medicine and Pharmacy, 700115 Iasi, Romania; 4 Center for Advanced Research and Development in Experimental Medicine (CEMEX), “Grigore T. Popa” University of Medicine and Pharmacy, 700115 Iasi, Romania; 5 Department of Pediatric Surgery, “Grigore T. Popa” University of Medicine and Pharmacy, 700115 Iasi, Romania

**Keywords:** neurodegenerative, renin, angiotensin (1–7), brain RAS, Alzheimer’s, Huntington’s, multiple sclerosis

## Abstract

Brain neurodegenerative diseases (BND) are debilitating conditions that are especially characteristic of a certain period of life and considered major threats to human health. Current treatments are limited, meaning that there is a challenge in developing new options that can efficiently tackle the different components and pathophysiological processes of these conditions. The renin-angiotensin-aldosterone system (RAS) is an endocrine axis with important peripheral physiological functions such as blood pressure and cardiovascular homeostasis, as well as water and sodium balance and systemic vascular resistance—functions which are well-documented. However, recent work has highlighted the paracrine and autocrine functions of RAS in different tissues, including the central nervous system (CNS). It is known that RAS hyperactivation has pro-inflammatory and pro-oxidant effects, thus suggesting that its pharmacological modulation could be used in the management of these conditions. The present paper underlines the involvement of RAS and its components in the pathophysiology of BNDs such as Parkinson’s disease (PD), Alzheimer’s disease (AD), multiple sclerosis (MS), Huntington’s disease (HD), motor neuron disease (MND), and prion disease (PRD), as well as the identification of drugs and pharmacologically active substances that act upon RAS, which could alleviate their symptomatology or evolution, and thus, contribute to novel therapeutic approaches.

## 1. Introduction

The reason for initiating this study was based on two premises: neurodegenerative (NDG) diseases are especially characteristic of later periods in life, with well-known exceptions, and RAS medication is extremely widespread in the same age groups. Given these significant overlaps, and in the context of repurposing becoming an increasingly common technique [1,2], we considered it useful to observe how NDG diseases are influenced by RAS and whether pharmacological modulation of RAS could have beneficial effects on the evolution and outcomes of BNDs.

Prorenin can be found in juxtaglomerular (JG) cells, which are specialized cells from within the afferent arterioles of the kidney. By activating JG cells, prorenin is cleaved into its active form called renin. It, in turn, will act on angiotensinogen (Angt), which is produced in the liver and can be found in plasma. This will lead to the formation of angiotensin I with a decapeptide structure [3]. Angiotensin (Ang) I is converted into Ang II through the action of angiotensin-converting enzyme 1 (ACE1), also called kininase II, an enzyme that is also involved in the degradation of bradykinin (BK) [4,5,6]. Accumulation of BK leads to a series of side-effects specific for ACE inhibitor therapy (e.g., cough and angioedema). The effects of RAS are mediated by Ang II following the stimulation of Ang II type 1 (AT_1_) receptors: sodium and water retention, vasoconstriction, and aldosterone synthesis [7]. Other components of RAS are angiotensin-converting enzyme 2 (ACE2), angiotensin fragments (Ang (1–7), Ang III, Ang IV), as well as different receptors (AT_2_, AT_4_, MAS) [8].

It is known that RAS hyperactivation generally has deleterious effects on neurons in culture and in vivo, mediated by its pro-inflammatory and pro-oxidant actions, and achieved mainly through the AT_1_ receptor (AT_1_R), but also intracellularly or through the multitude of metabolically active peptide fragments of Angt, Ang I or II that are formed via the action of tissue or circulating enzymes [5]. Another important aspect is that RAS itself has very efficient self-modulation modes inside AT_2_R, Ang (1–7), Ang IV/AT_4_R, etc. [9]. However, in many cases, patients receive ACE inhibitors (ACEIs) or angiotensin receptor blockers (ARBs) in the context of hypertension and heart or kidney disease. Modulating RAS in the context of BNDs needs to be properly addressed by healthcare providers.

The present paper seeks to summarize the implications of RAS and its components during the onset, evolution, and termination of BNDs and identify drugs and pharmacologically active substances that modulate RAS that could alleviate the symptomatology or evolution of BNDs.

## 2. Evidence of the RAS in the CNS: Presence of mARN, tARN, AT_1_ and AT_2_ Receptors, Mas Receptors, Ang II, Ang (1–7), and Ang IV

RAS is an endocrine axis which has important peripheral physiological functions (blood pressure and cardiovascular homeostasis, water and sodium balance, and systemic vascular resistance) [5]. In addition to the systemic action of RAS, recent research highlights its paracrine and autocrine actions in many tissues, including the central nervous system [10]. The complex is located intracellularly and is involved in various intracellular processes [11].

The impermeable nature of the blood-brain barrier (BBB) for RAS components through brain regions imposes a separation between tissue components from intraneuronal ones. However, a multitude of studies demonstrate the existence of a RAS system of the CNS, complete with enzymes, precursors, and receptors of all kinds, and even peptides and/or receptors that are no longer found in other tissues, such as Ang IV/AT_4_R [12,13,14].

It is well known that the systemic RAS is an endocrine system responsible for the regulation of homeostasis and the brain-localized RAS is involved in cognitive processes, such as memory and learning. These two types of RAS (systemic and brain) interact with one another [4,9,15,16]. Most brain regions do not have access to peripheral RAS, although the forebrain pathway allows peripheral access of RAS components, whereas the BBB restricts the peripheral RAS components from reaching the brain [5]. This pathway connects the circumventricular organs (CVOs), which are considered brain regions lacking the BBB, to the medial preoptic, supraoptic, and paraventricular nuclei. The brain is responsive to both circulating Ang II acting on its receptors found in BBB-deficient CVOs, as well as to centrally generated Ang II [17].

Stimulation of the renin/prorenin system has inhibitory effects on cognition [18], by activating the expression of Ang receptors (Ang Rs). Prorenin has its own receptors in the brain (PRR), although a number of authors believe that the entire cerebral renin/prorenin system is controversial [19].

Perivascular astrocytes are one type of glial cell that are important mediators of BBB formation, being an anatomical intermediary between neuronal circuitry and blood vessels [20]. Their neuroprotective effects are due to the formation of a scar-like perivascular barrier surrounding the demyelinated area, and thus limiting the influx of leukocytes into CNS parenchyma [21].

These cells located almost everywhere in the brain are a main source of the Ang II precursor, Angt [22]. Neurons are also capable of producing Angt, which is further converted to effector Ang II and other active angiotensins through the influence of intracellular renin and prorenin [5].

It was confirmed that a decreased level of Angt-expressing astrocytes was observed in lesions, as well as in Angt-deficient mice [23]. Both situations were correlated with a reduced expression of the tight junction (TJ) protein, occludin, at the level of the BBB, along with the accumulation of endogenous serum proteins into perivascular and parenchymal areas of the brain. Ang II is thus considered to upregulate the expression of occludin and strength of the BBB. Decreased production of Ang II can contribute to BBB dysfunction in patients with MS, AD, and other diseases [24].

Angt is found predominantly in the subfornical organ, paraventricular nucleus, nucleus of the solitary tract, and rostral ventrolateral medulla, but also in the ventrolateral medulla, hypoglossal nuclei, thalamus, hypothalamus, forebrain, and brain stem [25,26].

A major component of RAS in the nervous system is ACE. It is identifiable everywhere in the CNS, mainly due to its association with vascularization, but there is countless evidence that it exists and functions even in neurons and glia [27]. ACE2 is the variant that transforms Ang II into Ang (1–7) as well as Ang I in Ang (1–9), both with neuroprotective effects [28].

Brain RAS has a different importance compared with systemic RAS, being involved in cerebroprotection, stress, depressive disorder, and memory consolidation [18,29].

AT_1_R, AT_2_R, and MasR are located on the cell membranes, mitochondria, and nuclei [30]. RAS receptors are widespread in both neurons as well as in the three types of glial cells mentioned above. It has been shown that neurons have four main Ang Rs located in mitochondria and nuclei, such as AT_1_R, AT_2_R, MasR, and AT_4_R [30,31]. Type 1 and type 2 Ang Rs can be found along the spinal cord, as well as in different areas in the brain [32]. AT_1_R is expressed in the CNS by astrocytes, microglia, and even by neurons, particularly DA neurons. AT_1_R is expressed intracellularly, inside the nuclei and its membrane, as well as in the endoplasmic reticulum membrane [33].

Although Ang II/AT_1_R signaling has neurotoxic effects and can cause cognitive impairment due to vasoconstriction, inflammation, oxidative stress, proliferation, and cell death, Ang II/AT_2_R and Ang (1–7)/MasR promote neuroprotection and counteract the effects of AT_1_R activation. AT_4_Rs are located in the sensory and cognitive neuronal regions and are involved in learning and memory processes and mediate acetylcholine (Ach) and dopamine (DA) release [25].

Gurley et al. observed that plasma concentration of Ang II was almost three-fold higher than in controls (wild-type (WT) littermate mice) after acute infusion of Ang II in ACE2-deficient mice, suggesting the in vivo role of carboxypeptidase as a functional component of RAS in metabolizing Ang II [34].

Ang I can be converted by enzymatic cleavage in the presence of aspartyl aminopeptidase (ASAP) converts it into Ang (2–10) aminopeptidase A (APA) hydrolyses Ang II into Ang III by removing the N-terminal Asp [35,36]. The resulting Ang III is a vasoconstrictor peptide and can subsequently be converted to Ang IV in the presence of aminopeptidase B or aminopeptidase N. Cleavage of Ang IV leads to the synthesis of Ang (3–7). Ang II can also be converted to Ang (1–7) and this can be converted to Ang (2–7) [29,37,38].

**AT_1_Rs** are located in the hippocampus, cortex, and basal ganglia. Ang binds to this type of receptor and induces conformational changes of the receptor proteins with the activation of a G protein, followed by mediation of signal transduction. AT_1_Rs are G protein-coupled receptors that, in the presence of Ang II, activate Gαq protein signaling followed by dissociation of the Gαq domain. This subunit activates phospholipase C (PLC) involved in the production of diacylglycerol (DAG) and inositol triphosphate (IP_3_) through the metabolism of phosphatidylinositol biphosphate (PIP_2_). The binding of IP_3_ to endoplasmic reticulum receptors promotes calcium release into the cytoplasm [5,29,39].

Chronic activation of AT_1_Rs at the neuronal level causes cognitive impairment, inflammation, and cell death [14].

**AT_2_Rs** share structural characteristics with members of the 7-transmembrane receptor family, belonging to the family of G protein-coupled receptors. They are widespread in the brain, showing very high densities in structures such as the amygdala, thalamus, putamen, and tegmental area [29]. The pro-inflammatory and pro-oxidant effects of Ang II mediated by AT_1_Rs are counteracted by activation of AT_2_Rs by Ang II and Mas Rs by Ang (1–7). However, an increase in AT_1_R expression and decrease in AT_2_R expression have been observed in the aging brain, which could contribute to the increased vulnerability of neurons [14]. Numerous studies have shown that AT_1_R and NADPH-oxidase expression can be decreased by activating AT_2_R, leading to decreased inflammatory responses [40].

The vascular and direct nerve cell effects of Ang II are difficult to separate, with numerous studies incriminating Ang as a causative factor in various neurodegenerative conditions and/or memory impairment [41]. Several studies have linked the accumulation of β-amyloid (A-β) and tau (τ) protein as a result of increased AT_1_R activation by Ang II [42]. Intracerebroventricular Ang II administration reduces cerebral blood flow, inhibits potassium-mediated acetylcholine release, impairs spatial memory, and induces oxidative stress [38].

Ang II via AT_1_Rs mediates vasoconstrictor, pro-oxidant, and inflammatory actions, this peptide together with the converting enzyme being increased with the aging process [14,43].

Downstream signaling of Ang II AT_1_Rs requires the involvement of events such as the activation of enzymes that may lead to reactive oxygen species (ROS) synthesis, inflammation, and apoptosis [17]. This enzyme via AT_1_R activates nicotinamide adenine dinucleotide phosphate (NADPH) oxidase, which mediates oxidative stress through the formation of reactive oxygen species (ROS) and inflammatory processes, with implications in NDG diseases [14,39].

Some studies argue that brain RAS is involved in the degradation of dopaminergic transmission and the excessive stimulation of Ang II AT_1_Rs exacerbates dopaminergic cell death. Vulnerability of dopaminergic neurons may be driven by increased oxidative and pro-inflammatory stress associated with the aging phenomenon, a risk factor in NDG disorders such as AD [30]. Ang II influences tumor necrosis factor-α (TNF-α) and transforming growth factor-β (TGF-β) signaling and inhibits potassium-mediated acetylcholine (Ach) release. Pharmacological inhibition of Ang II may antagonize the effects of scopolamine on cognitive impairment [42].

Stimulation of AT_2_ receptors causes opposite results to those produced by the binding of Ang II to AT_1_Rs. Therefore, vasodilation occurs and inhibits increased proliferation [44].

**Ang (1–7)** is a heptapeptide with a protective role, capable of counteracting cellular senescence and inflammation as hallmarks of vascular aging. This peptide has actions opposite to those produced by Ang II by binding to the G protein-coupled Mas R [43]. In the brain, Ang (1–7) has been shown to counteract the pro-apoptotic effects of Ang II [15], stimulating cerebral angiogenesis and proliferation of cerebral endothelial cells, which has been confirmed in animal models. Ang (1–7) perfusion for 4 weeks in rats resulted in improved oxygen and blood supply, stabilizing brain energy status with reduced neuronal consequences. Regarding its neuroprotective role, Ang (1–7) also acts through other Mas/nitric oxide synthase (eNOS)-dependent mechanisms, modulating oxidative stress and inflammatory response [43,45].

The role of Ang (1–7) in cognitive processes such as memory and learning and in stress processes, with localization in central brain areas such as the amygdala and hippocampus, has been reported [46]. The ACE2/Ang (1–7) neuronal axis/Mas R generates anti-inflammatory and antioxidant effects, with Mas R facilitating cell survival through its activation by Ang (1–7). High levels of ACE_2_ cause Ang (1–7) synthesis; in other words this enzyme is responsible for enhancing cognitive processes and its deficiency leads to pro-oxidant effects [15].

**Ang IV**, a metabolite of Ang II and its receptor AT_4_, is a membrane protein. It is known as insulin-regulated aminopeptidase (IRAP) type II, localized in the brain on neurons in the hippocampus, cortex, and basal ganglia, and is not a G-protein coupled receptor [5,47]. In an AD mouse model, activation of brain AT_4_Rs antagonized cognitive impairments caused by A-β pathology, suggesting that Ang IV and its analogues could be considered as therapeutic targets [47]. Metabolism of Ang II to Ang IV is the most likely underlying cause of beneficial effects, such as improved cognitive processes, which have been observed in animal model testing [48].

## 3. Renin-Angiotensin-Aldosterone System and Parkinson’s Disease

PD is characterized by the degeneration of DA neurons in the substantia nigra (SN) of the midbrain, leading to subsequent motor symptoms, such as rigidity, tremor, ataxia, and postural instability [49].

There are several mechanisms presumed responsible for the destruction of dopaminergic neurons in the SN: oxidative stress, neural inflammation, and mitochondrial dysfunction [50]. DA neurons are highly susceptible to damage in relation to high ROS levels [14]. Neural inflammation is evidenced in PD degeneration by the high levels of inflammatory cytokines. Activated microglial cells lead to dopaminergic cell death by phagocytosis and increased ROS production [51].

Brain RAS has been lately reported in influencing dopaminergic regulation, neurotransmission, and neuron survival [52].

A significantly higher level of Ang II in the CSF or brain tissues was observed in PD animal models, as well as patients with this disease. Ang II is secreted by glial cells (microglia, astrocytes, and oligodendrocytes) in regions responsible for cardiovascular functions and in other brain regions. It plays an important role in memory, anxiety, bipolar disorder, and PD [53]. The level of AT_1_R was also increased in the SN of PD rats and overactivation of the brain Ang II/AT_1_R axis contributed to the progression of PD [54]. Mitochondrial ATP-dependent potassium channels (K_ATP_) induce dopaminergic neuronal loss, stimulate the superoxide-induced damage, and increase the inner mitochondrial membrane potential induced by Ang II administration. All these aspects can be stopped or at least reduced by inhibiting Ang II stimulation by ARBs [55].

The coupling of Ang II to AT_1_R leads to inflammation, increased ROS production, and activation of the NADPH complex. Its overactivation determines an increase in DA release, as well as neuroinflammatory and neurotoxic effects in the brain. The Ang II/AT_1_R axis has inhibitory effects on GABA and excitatory effects on glutamate. Thus, it is suggested that activating type 1 receptors plays an important role in motor deficits in PD through the indirect pathway of the SN [56]. Large studies suggest that using RAS inhibitors may be associated with a reduced risk of PD. However, a statistically significant risk reduction in PD incidence was observed after administration of ARBs, but not in the case of ACEI use [57].

The neuroprotective effects due to the binding of Ang II to AT_2_R improve neural growth as well as cognitive processes, learning, memory. This is caused by activation of nitric oxide (NO) production [25]. Low levels of Ang II are linked to an increase in the severity of depressive symptoms [58].

AT_1_R has pro-apoptotic activity in DA neurons, either through oxidative stress mediated by NADPH or through apoptotic signaling of the mitochondria [54]. In the DA neurons of SN, Ang II enhances the expression of mRNA and AT_1_R protein, inducing their overactivation. AT_1_R activates a mitogen-activated protein kinase (MAPK) cascade initiated by the protein kinase C (PKC), which also activates the NADPH complex, the main source of ROS production in the cell [59].

Ang II/AT_2_R activation may be a pathway for a series of neuroprotective effects on cerebral tissue. AT_2_R increases NO production, stimulates neurite growth, and has beneficial effects on memory, learning, and cognition [25]. The Ang II/AT_1_R axis is responsible for the dopaminergic neuronal loss and reduction of 70% of tyrosine hydroxylase neurons restored with candesartan (Ang II-receptor inhibitor) and azilsartan in rotenone, 1-methyl-4phenyl-1,2,3,6-tetrahydropyridine (MPTP), and 6-hydroxydopamine (6−OHDA) PD mice models [54].

A large study in Korea clearly demonstrated that ARB only had protective effects on the development and evolution of PD, whereas the statistical significance of long-term ACEI use remained elusive, a phenomenon attributed by the authors to the increased BBB permeability of the ARBs [60].

## 4. Renin-Angiotensin-Aldosterone System and Alzheimer’s Disease and Other Memory Disorders

AD is a neurodegenerative disorder characterized by abnormal changes, with progressively severe evolution leading to cognitive decline with memory loss. It is accompanied by behavioral disorders as well as functional deterioration of some organs [4,29,47].

Pathologically, the brains of patients with Alzheimer’s dementia are affected by extraneuronal A-β plaques, intraneuronal tangles formed by hyperphosphorylated τ proteins associated with microtubules, neuroinflammation, brain atrophy, especially in the hippocampus and neocortex, and BBB damage [4,61,62]. The induction of pathological processes in AD are mainly due to the accumulation and deposition of A-β in certain brain areas, with A-β plaques being major causes of neurotoxicity [38].

Various studies have shown that AT_1_R blockers and ACEIs inhibit CNS inflammation [14,39]. Preclinical studies suggest that ARBs regulate enzymes involved in the catabolism and elimination of A-β peptides, thus demonstrating the positive effects of blockers in preventing vascular damage caused by elevated levels of A-β (Table 1 and Table 2) [63].

Stress-inducers increase brain levels of Ang II, thus having an important role in AD. This hypothesis is supported by numerous studies that have reported that ARBs, such as losartan, olmesartan, candesartan, and valsartan, improved memory and other cognitive parameters; this has been observed in both animal models and patients with AD [29]. Ang II AT_1_ receptor blockers known as sartans have been recognized to have anti-inflammatory, neuroprotective, and neurorestorative effects in experimentally induced brain injury [67]. In a preclinical study conducted on primary cell cultures isolated from 8-day-old Sprague Dawley rat pups, the excitotoxicity of glutamate, a pathogenic factor in AD, and the effects of candesartan on glutamate were evaluated. The findings of this study revealed that candesartan prevented glutamate-induced changes in gene expression, but its effects were not related to Ang II AT_2_ receptor stimulation because AT_2_Rs are not expressed in rat primary cerebellar granule cells (CGC) in vitro. The results of this study argue for the inclusion of ARBs as a drug of choice for early cognitive impairment [68,69]. The anti-inflammatory effects of ARBs are due to the reduction in pro-inflammatory factors in the cerebral circulatory system, as well as the increase in cerebral flow that antagonizes neurodegeneration induced by cerebral ischemia [39]. Moreover, it has been shown that these drugs promote neuroprotection in numerous animal (rodent) models of AD by improving cognitive parameters (Table 2) [69].

In an amyloid precursor protein (APP)/PS1 transgenic mouse model of AD treated with intranasal losartan at a dose of 10 mg/kg every other day for 2 months, A-β plaque formation decreased 3.7-fold [83].

In another preclinical study based on cultures of cortico-hippocampal neurons obtained from the Tg2576 AD mouse model, another blocker, valsartan, was shown to prevent spatial memory deficits by attenuating oligomerization of AD-type A-β peptides [84].

The anti-Alzheimer’s effect of telmisartan was demonstrated in an ovariectomized hyperglycaemic rat model, causing decreased expression of Ang I and II receptors in the hippocampus, improving cognitive impairment and reducing A-β and τ protein levels [85].

ACEI therapy improved cognitive performance and reduced the risk of vascular dementia. Moreover, cognitive function was stabilized in patients with mild cognitive impairment [86]. According to a meta-analysis, both ACEIs and ARBs were found to have beneficial effects on cognitive decline, with ARBs being the most effective in preventing dementia [87]. Treatment with ACEIs in AD was associated with improvement in cerebral blood flow, anti-inflammatory activity by decreasing inflammatory cytokine activity, stimulation of cholinergic neurotransmission by increasing acetylcholine levels, and reductions in oxidative stress [87,88].

Treatment with captopril and perindopril, which are examples of ACEIs that cross the BBB, reduced A-β levels. Captopril’s inhibition of the enzyme converting Ang I to Ang II prevented AD-related decreases in transcript levels of several hippocampal genes involved in functions related to cognitive processes. Other studies showed that central ACE inhibition demonstrated neuroprotective activity, due to the suppression of pro-inflammatory microglia, attenuation of oxidative stress, and astrocyte activation (Table 3) [72].

A recent clinical trial comparing the effects of ACEIs with those of ARBs when used in asymptomatic and symptomatic patients at different stages of cognitive impairment found that ARBs may be more protective against cognitive decline than ACEIs. Use of ARBs vs. ACEIs was associated with slower accumulation of A-β in patients with normal cognitive status. However, in those with AD, the use of ARBs vs. ACEIs did not show different rates of A-β accumulation [63].

Aliskiren is a renin inhibitor that blocks Ang I synthesis from Angt through the intracellular modulation of gene expression after its binding to the nuclear PRR. Plasma renin activity remains suppressed because Angt cleavage is blocked, although aliskiren leads to increased plasma renin concentrations [6,95].

Cognitive impairment can be mitigated by aliskiren, which directly inhibits renin [96]. Building on this idea, another study on cortical neurons cultured in vitro showed that the neuroprotective effects of aliskiren were due to neutralizing A-β toxicity [97] (Table 4).

## 5. Renin-Angiotensin-Aldosterone System and Multiple Sclerosis

Multiple sclerosis (MS) is an autoimmune disease of the CNS that affects both gray and white matter tracts, as well as the brain stem and basal ganglia [99,100].

The neurological impairment of MS includes demyelination, axonal or neuronal loss, astrocyte gliosis, as well as oligodendrocyte degeneration in the CNS [101]. The initial inflammatory relapsing phase leads to astroglia proliferation [102] and neurodegenerative phase [103,104,105]. Focal inflammatory lesions within MS plaques are due to the involvement of both adaptive and innate immunity [106]. In addition to their basic role in protecting from infection, the lymphocytic inflammatory infiltrates negatively influence MS pathology through the activation of microglia and macrophages, as well as breakdown of the BBB [107]. B-cells are important trigger factors of sustained inflammation due to their stimulatory effect of T-cell responses [106,108].

Activated astrocytes by T-cells have a dual role during the development of the disease. They promote neurotoxicity in most areas where myelin sheaths are damaged, by secreting oxygen and nitrogen radical species, glutamate, and ATP [23]. Their local activation has a negative effect at major sites of progressive MS and leads to tissue destruction and neurological impairment [109]. They modulate BBB permeability and CNS inflammation due to the multifocal production and infiltration of cytokines [24,110].

The influence of RAS in microglial polarization is the consequence of two opposite effects. Thus, under pathological conditions, AT_1_R and AT_2_R are upregulated and activate microglia. The effects of nuclear AT_1_R that leads to pro-inflammatory/classically activated microglia (M1 substate) are counteracted by the opposite RAS arm represented by Ang II/AT_2_R that leads to anti-inflammatory/alternatively activated microglia (M2 substate) [14,111].

RAS is also spread in oligodendrocytes where it triggers opposite effects depending on the type of receptors. Whereas AT_1_R activation promotes demyelination, AT_2_Rs lead to a re-myelination process with positive consequences in MS pathology [73]. A study by Lee et al. pointed out the presence of higher anti-AT_1_R antibodies titers in MS patients compared with those with stable relapsing-remitting or progressive MS, thus being correlated with recent disease activity [112].

Another conclusive aspect represents the involvement of RAS in immune activity. Accumulated evidence has demonstrated that AT_1_ receptors are located on immune cells, such as macrophages, T-cells, natural killer (NK), or dendritic cells [78]. By upregulating the concentration of pro-inflammatory markers in the brain (IL-6, IL-8, and TNF-α) and increasing oxidative stress, Ang II is reported to play a key role in the evolution of many autoimmune diseases, including MS, rheumatoid arthritis, or systemic lupus erythematosus [113]. It effects the Th1/Th2 cytokine response in MS, suggesting a possible shift to Th1 via stimulation of AT_1_Rs [114].

Recent studies showed that brain RAS components were altered intrathecally in the pathogenesis of MS. Kawajiri et al. described a strong decrease in CSF Ang II levels of 21 patients suffering from relapsing-remitting MS (RRMS) compared with control. This indicates that RAS may be involved in abnormal neuronal lesions and repair processes in MS [115]. The researchers conducted another study on 20 patients with RRMS without the anti-aquaporin-4 antibody and reported a supplementary decrease in ACE2 level, another important regulator in RAS. This homologue of ACE cleaves Ang II into Ang (1–7), which counterbalances the actions of Ang II. It was suggested that upregulation of ACE levels and downregulation of ACE2 levels were involved as compensatory mechanisms of the decrease in the level of Ang II [116].

Another study on a large cohort of 438 untreated patients with MS observed that age-adjusted CSF ACE levels were modestly decreased in purely relapsing and chronic progressive MS patients when compared with a control group consisting of 276 patients with non-inflammatory neurological disorders. Additional analysis revealed no significant difference in CSF ACE levels between currently relapsing and currently stable MS patients. A possible mechanism is the loss of perivascular astrocytes during the course of the disease with diminished intrathecal ACE release [117].

Other studies point out increased levels of RAS components in serum samples from MS individuals. For example, Constantinescu et al. reported elevated serum ACE activity in 17 of 75 patients with MS compared with 31 healthy controls, suggesting its involvement as an indicator in disease monitoring and therapeutic efficiency [74]. In another study, the authors showed that a 21-day treatment with captopril ameliorated the clinical course of monophasic experimental autoimmune encephalomyelitis (EAE) in Lewis rats, suggesting the important role of the RAS in the autoimmune inflammation of the CNS. This effect was in part due to a decreased responsiveness of T-cells both to antigens and mitogen [118]. Ottervald et al. analyzed CSF from 76 patients with SPMS using proteomic techniques and showed that Angt level was significantly higher (three-fold) when compared with 36 control individuals [119].

Some research results that were not consistent with the above-mentioned studies have been identified. For example, Razazian et al. analyzed serum ACE levels in 30 patients diagnosed with MS in a pilot study and did not find any significant differences compared with 30 healthy controls. These inconsistent results were explained by the authors to be probably due to the difference between serum and CSF properties, ACE activity and ACE levels, or differences between the laboratory techniques used [120].

An upregulation process of RAS components in the brain lesions of MS patients was observed in a report conducted by Platten et al. regarding the functional role of RAS components during autoimmune demyelination in EAE models. SJL/J female mice suffering from EAE immunized with proteolipid protein 139–151 peptide (PLP p139–151) were pretreated with ACE inhibitor lisinopril or AT_1_R antagonist candesartan. It was shown that ACE inhibitors or AT_1_R antagonists ameliorated the pathogenic condition by suppressing the autoreactive inflammatory Th1 and Th17 cells, as well as by increasing regulatory CD4+ FoxP3+ T-cells in the CNS. These aspects suggest that RAS is involved in driving autoimmunity in both MS and EAE models [121].

Stegbauer et al. investigated the role of the RAS in myelin oligodendrocyte glycoprotein (MOG)-induced EAE in C57BL/6 mice. An increase in serum renin activity and renin mRNA expression was observed in the spinal cord and spleen on the 31st day after immunization. Moreover, application of the renin inhibitor, aliskiren, as well as preventive or therapeutic treatment with the ACE2 inhibitor, enalapril, or with AT_1_R antagonist, losartan, improved the pathological course of MOG-EAE [80].

Lanz et al. provided evidence that AT_1_R inhibitors, such as candesartan, potently decreased TGF-β signaling in the brains and spinal cords of mice with chronic-progressive MOG-induced EAE, which led to attenuated severity of the disease. The immune response was reduced via a pathway involving the TGF-β-activating protease thrombospondin-1 (TSP-1) [122]. However, the baseline of TGF-β signaling was not altered, suggesting that other molecules were responsible for this process. It is known that Ang II is responsible for TGF-β overproduction in the brain and spinal cord during the onset of EAE [123].

Another study investigated the potential anti-inflammatory and neuroprotective effects of a 4-week treatment with AT_2_R agonist, Compound 21 (C21), in MOG-EAE C57BL/6 mice. It was found that C21 ameliorated neurological deficits through a reduction in demyelinated areas, T-cell infiltration, and the number of resident and activated microglia in the spinal cord. Complementary in vitro studies in aggregating rat brain cell cultures with lipopolysaccharide (LPS)/interferon-γ (IFN-γ) confirmed that AT_2_R activation protected from demyelination and microglial activation, as well as promoted remyelination and attenuated cytokine and NO release from microglia [73].

Guo et al. investigated the effects of candesartan on optic neuritis in an EAE mouse model of MS. Optic neuritis is an acute inflammatory disorder of the optic nerve that is associated with RRMS [124]. It was shown that candesartan ameliorated inflammatory-mediated degeneration of the retina and optic nerve as well as in the spinal cord through the inhibition of innate immune responses in astrocytes. In addition, researchers found that candesartan suppressed TLR4 expression in astrocytes that was increased by Ang II via the nuclear factor (NF)-κB pathway [125] (Table 5).

Modulation of RAS components may influence MS pathology through the modulation of the autoimmune response.

## 6. Renin-Angiotensin-Aldosterone System and Huntington’s Disease

HD, also termed Huntington’s chorea, is an autosomal dominant neurodegenerative disorder with a prevalence of 10.6–13.7 per 100,000 individuals in Western countries [137]. HD is caused by an abnormal expanded repeat of CAG trinucleotide in the huntingtin (HTT) gene, which leads to the formation of mutant huntingtin protein (mHTT), a key player in the pathogenesis of the disorder [137,138,139,140].

mHTT has been shown to misfold and accumulate as aggregates, thus leading to alterations in a series of cellular functions. mHTT-mediated oxidative stress must also be mentioned as an important component of HD. There is evidence that damage done to the mitochondrial DNA by oxidative stress is involved in the pathogenesis of HD; pathways that determine oxidative stress in HD include elevated nicotinamide adenine dinucleotide phosphate oxidase (NOX) activity, activation of the antioxidant defense system, as well as oxidation of mitochondrial enzymes [141].

Mitochondrial dysfunction is another hallmark pathogenic mechanism of HD. There are several causes of the mitochondrial dysfunction: reduced activity of mitochondrial respiratory chain complexes, deficits in the handling of Ca^2+^, increased production of reactive oxygen species, increased mitochondrial-fragmentation, and an accumulation of impaired mitochondria, which could be due to inefficient functioning of the degradation systems [142].

Other important pathways are linked to DA and glutamate. These can interfere with inhibitory or excitatory processes that take place in the basal ganglia, and it seems that the neurodegenerative processes characteristic of this disease can be due to an excess of glutamate. This leads to excitotoxicity, and finally, cell death [143].

Regarding symptoms characteristic of this disease is the triad of motor, psychiatric, and cognitive disturbances [144,145,146,147].

Considering that current therapeutic options for HD are symptom-oriented, discovering other mechanisms and systems involved in the pathogenesis of the disease is an important goal for researchers.

RAS is involved in different functions of the brain, including behavior, cognition, and motor control. It is linked to NDG diseases, with Ang II being considered a key component; it is involved in neuronal death through oxidative stress, inflammatory responses, and apoptosis [9]. These effects were confirmed in animal models of different NDG diseases, such as PD [148]. Thus, studying RAS may shed light on other NDG diseases, such as HD, allowing for alternative management strategies.

Studies have reported that the blockage of AT_1_ and AT_2_ receptors with compounds such as losartan and PD-123177, as well as ACE inhibition through the use of captopril, reduced oxidative stress in the hippocampus of rats, an effect that could be explained by the central inhibitory effect of Ang II. This could lead to a possible new therapeutic approach, considering that oxidative stress may play a role in the pathogenesis of HD. Another important finding was the association between higher levels of ACE2 and higher scores on tests that evaluate verbal fluency, suggesting that higher ACE2 levels are correlated with better verbal function [149,150] (Table 3).

A study conducted by Hariharan et al. evaluated the protective potential of trandolapril in a 3-nitropropionic acid (3-NP) rat model of HD. Trandolapril is a prodrug of trandolaprilat, which is an ACE inhibitor. It is highly lipophilic and centrally active, with a high inhibitory potency of ACE [151]. Through the inhibition of Ang II, trandolapril exerts neuroprotective effects and lowers oxidative stress by reducing mitochondrial dysfunction. Pre-treatment with trandolapril improved a series of symptoms that are specific for HD, restoring body weight loss in 3-NP rats and improving motor incoordination. Trandolapril contributed to better cognitive performance in behavioral tasks and restored several respiratory chain enzymes which were found to be depleted by 3-NP. The authors suggest that the effects of ACE inhibition in HD may be a starting point for determining the exact mechanisms that underlie its neuroprotective effects. This, in turn, could lead to different therapeutic approaches for a disease that currently has limited management options [152].

Other researchers have shown that the use of ARBs, such as losartan and candesartan, could protect against neuronal cell death caused by the administration of MPTP in rats. Although this is a specific PD animal model, the fact that DA pathway impairment is also observed in HD could mean that ARB therapy may have utility in the management of this disorder as well. It was shown that candesartan led to the inhibition of Ang II on DA cell death, the effect being due to candesartan’s potency as a AT_1_ blocker as well as its ability to cross the BBB, thus inhibiting the central effects of Ang II [149].

Sengul et al. studied the effects of ACEIs (captopril, ramipril, and perindopril) on the glutamate pathway, which is known to be involved in the generation of neurotoxic effects. Neurotoxicity was induced by administering glutamate in newborn rat cerebral cortex cells. The authors concluded that ACEIs could be beneficial in reducing glutamate-induced toxicity, either by reducing free radicals or by increasing antioxidant protective mechanisms [129].

In a review conducted by Machado et al., striatal cells expressing mHTT were sensitive to Ang (1–7), but Ang II had minor effects in the same cells. This may be explained by the reduced expression of AT_1_R [153]. In another study by Imamura et al., the effects of Ang II on the R6/2 mouse model of HD were evaluated. It seems that Ang III determined some positive outcomes, such as reduced body weight decline, prolonged lifespan, and recovery of striatal-neuron DNA damage, while also improving dendritic length and restoring dendritic spine density. It did not, however, improve motor functions, nor did it reduce the number of inclusion bodies of mHTT in the striatum. These findings suggest that though Ang III inhibited the interaction between mHTT and its target, it did not suppress protein aggregation [154].

De Mello et al. have shown that mHTT-expressing striatal cells are sensitive to Ang (1–7), with its effect being related to the MasR that can be found in areas such as the amygdala, hippocampus, forebrain, olfactory bulb, piriform cortex, thalamus, and portions of the hypothalamus. Studies have shown that patients with HD had reduced activity of ACE in brain regions specific to HD pathogenesis, such as the caudate nucleus, putamen, and globus pallidus. These findings suggest that the ACE/Ang II/AT_1_R axis is reduced in HD patients, whereas the ACE2/Ang (1–7)/MasR axis in mutant neurons is predominantly activated, suggesting the involvement of RAS in the pathogeny of HD [9].

Another study by Steventon et al. highlighted the potential negative impact of hypertension on patients with HD and the positive outcomes of antihypertensive treatment. Although this study focused on an array of antihypertensive drug classes, and not only on drugs targeting the RAS, it demonstrated that hypertensive HD patients who did not receive treatment for hypertension had reduced functional capacity, as well as worse cognitive, motor, and depressive symptoms compared with normotensive and hypertensive HD patients. Thus, the study offered clues regarding the possible beneficial effects of antihypertensive drugs, which included the RAS inhibitors that are of interest to this review. The medication resulted in a reduction of disease severity in HD patients with hypertension, suggesting the potential involvement of antihypertensive drugs in the management of the disorder [155].

Other findings included an attenuation of cognitive impairment following the administration of AT_1_R antagonists, sustaining the idea that antihypertensive drugs that target RAS may be beneficial in NDG diseases. However, further studies are required for evaluating their specific effects and mechanisms in HD patients [148].

## 7. Renin-Angiotensin-Aldosterone System and Motor Neuron Disease

MND is a generic name for several neurological pathological entities that progressively afflict motor neurons, reducing body motor abilities until invalidity and death. Specific symptoms include muscle atrophy, muscular spasms such as fasciculations, muscle spasticity, and/or hyperreflexia. The capability to walk, speak, swallow, or breathe is gradually lost.

Among the MND, we count:

- Lateral amyotrophic sclerosis (ALS);
- Progressive bulbar palsy;
- Primary lateral sclerosis;
- Progressive muscular atrophy;
- Spinal muscular atrophy;
- Kennedy disease;
- Post-poliomyelitis syndrome [156,157,158].

The end usually occurs after 3–5 years of evolution, most frequently due to respiratory failure. It is difficult to ascertain a direct link of any kind between RAS and this group of diseases. However, even though it is generally accepted that Ang II can initiate neuronal death and disease, it is acceptable to count the blocking of these actions among the strictly reduced means available to treat or palliate the effects of motor neuron diseases.

Several years ago, Japanese [116,159] and Taiwanese [160] researchers coincidentally discovered that the administration of pharmacological treatments that modulate the RAS may induce a more favorable evolution of lateral amyotrophic sclerosis (LAS). According to them, the cerebrospinal concentration of Ang II is negatively correlated with the presence and degree of evolution of MND.

Among the theories suggested by various researchers, one was the possible protection of neural tissue through the AT_2_R, due to its antioxidant and cell proliferation-stimulating actions. Another theory was the reduction in the amount of Ang II from the cerebral tissue, which would reduce the deleterious effects of AT_1_R stimulation. These hypotheses have been correlated with similar effects seen in patients with AD or PD [161,162,163,164,165]. The enhancement of glial and neuronal inflammation mediated by the AT_1_R, accompanied by microgliosis and the added stimulation of oxidative stress by extracellular and intracellular Ang II have been linked to MND [166,167].

Another hypothesis was the direct augmentation of neural protection through vitamin E (α-tocopherol), a known liposoluble antioxidant [94] (Table 3).

The inhibition of the glutamatergic stimulation was one of the other presumptive mechanisms of the protection induced by ACE blockers in other NDG afflictions [129].

Studies using neuronal cell cultures demonstrated that Ang II, in the presence of aldosterone, another component of the same physiological system, may have a deleterious effect on neurons, with possible astrocytic involvement. Inhibition of the aldosterone receptor with eplerenone [168] reduced the damaging effect on the neurons more than AT_1_R inhibition by valsartan. The deduction obtained from this seminal study was that astrocytes, stimulated by Ang II, produced more aldosterone, which had a damaging effect on neurons in culture. This effect, in turn, was inhibited by the addition of eplerenone [169] (Table 6).

Since 2002, it has been claimed that the administration of ARBs such as Olmesartan has beneficial effects on neuronal survival [175].

Another cohort study, presented elsewhere, demonstrated that the association of a long-term ARB treatment reduced the risk of PD by 44% [91]. In AD, researchers have proposed ARBs as pharmacological tools, among which valsartan seemed to have the best neuroprotective effect, candesartan had optimal effects for the reduction in neuroinflammation, and telmisartan was the best for reducing gliosis. ACEI also had beneficial effects [4]. Bearing in mind the reduced quantitative importance on the MND, compared with the other diseases, more studies should focus on MND. However, the lessons learned from other neurodegenerative diseases are also applicable in the investigation for novel treatment avenues of MND. Recently, a receptor called HGF/c-Met (hepatic growth factor/tyrosine kinase type I receptor) has been identified [176]. Molecular functional data from behavioral studies suggest that this is one and the same with the ligand/receptor system Ang IV/AT_4_ [75]. Two potent antagonists of this system have been synthesized, divalinal-Ang IV and norleual-Ang IV, together with an agonist Nle1-Ang IV. However, these have significant pharmacokinetic limitations, which make them yet unusable for therapeutic purposes (Table 2).

Another hypothesis involving AT_4_R is one that assimilates it with the IRAP receptor [177,178]. As such, a series of pharmacological modulators have been proposed, among which Dihexa (N-hexanoic-Tyr-Ile-aminohexanoic amide) significantly improved memory retention and reduced cognitive deficits; it is to be investigated in the therapy of motor dysfunctions [47,77].

In 2015, Lin et al. demonstrated a spectacular (57%) reduction of ALS in patients with chronic use of ACEI over 4 years [160]. However, their data were contradicted by later studies. In a recent study by Franchi et al., chronic administration of large doses of ACEI and ARB were not accompanied by a statistically significant improvement in the evolution of the NDG disease [179].

In a very large study, Pfeiffer followed 10,450 ALS diagnosed cases and 104,500 controls over 6 years to see if adjacent medication had any effect on the main disease after at least 6 years of treatment. More than 700 drugs were investigated, and it was determined that lisinopril was significantly associated with lowered ALS risk (to OR¼ 0.88) [180].

A very interesting paper tried to identify differentially expressed genes that occur in ALS. When identifying potential clusters of mutations that might increase the risk of neuronal death in ALS, the Ang genes, among many others, were detected as hub genes that could be targeted as novel therapeutic targets for ALS disease [181].

A computerized approach was also used for investigating the involvement of the RAS/ACE axis in the pathology of spinal muscular atrophy as an aggravating factor of respiratory failure in NDG diseases and their interaction with COVID-19. The involvement of ACE2 in the mediation of the SARS-CoV-2 penetration of host cells was considered; as a result, it was also assumed that reducing ACE2 activity reduced bradykinin degradation with significant cough and shortness of breath, a key functionality in the pathogenesis of COVID-19.

Other studies have demonstrated an interaction between the survival motor neuron 1 (SMN1) gene and the loss of function of the SMN protein, which is involved in ribonucleoprotein synthesis, intra- and intercellular trafficking of vesicles and organelles, and ACE/ACE2 expression [182,183].

## 8. Renin-Angiotensin-Aldosterone System and Prion Disease

The term was coined by Prusiner in 1982 and means “proteinaceous infectious particles” [184,185]. These are NDG diseases induced by the natural transformation of a neuronal natural membrane protein called PrP^C^ (cell prionic protein), a membrane glycoprotein that presents two helixes and two complex oligosaccharide chains coupled at the N-terminal head [186].

This is a normal protein, abundantly present within the neuronal cell membrane, anchored by a GPI (glycosyl-phosphatidyl-inositol) anchor and aggregated in lipid rafts. It is encoded by the PRNP gene, located on chromosome 20 in humans and on other chromosomes in various animal species [187]. Besides neurons, it has also been identified in glial cells, immune cells, epithelial cells, or endothelial cells. Its major implications appear to be in cell survival, especially against free radical aggression and apoptosis, as well as in cell adhesion [188]. However, there are situations in which PrP^C^ is internalized and metabolized in multiple ways, which seem to change its functions. Among the essential physiological functions of this protein are protection against apoptosis induced by lack of growth factors, protection against oxidative stress, and protection against endoplasmic reticulum stress, caused by the accumulation of misfolded/unfolded proteins in the endoplasmic reticulum [189].

PrP^C^ frequently interacts with a multitude of neuronal proteins, including nicotinic cholinergic receptors. They control their postsynaptic activation, which could explain the cholinergic manifestations induced by PRD [190].

Also, there is a very well-documented interaction of PrP^C^ with the τ proteins in AD and with α-synuclein, which could be responsible for some parkinsonian symptomatology in PRDs [191]. Similar to the evolution of PrP^C^ towards PrP^Sc^ in PRDs such as kuru, Creutzfeldt-Jakob, GSS, and fatal familial insomnia, all NDG afflictions show misfolding proteins, such as α-synuclein [192], A-β, APP, τ in AD, TDP-43 in ALS, HTT, and the amyloid protein [193]. These have been labeled “prionoids” and some attempts to mimic the disease by transferring these proteins in cell cultures or animal models have been successful in inducing neurotoxicity [194,195] (Table 6).

PRD includes Scrapie in sheep, bovine spongiform encephalopathy (mad cow disease), Creutzfeldt-Jakob disease in humans, and kuru disease. All of these are characterized by the transformation of the alpha helix structured PrP^C^ into the pathological form of the scrapie PrP^Sc^ protein, which has a beta-pleated structure. Characteristic of these diseases, contact between PrP^C^ and PrP^Sc^ will transform it into PrP^Sc^, thus producing two misfolded proteins. These in turn will interact with two others, which they will transform, and so on ad infinitum. Interestingly, in sporadic disease, the transformation can be spontaneous without the intervention of an external PrP^Sc^ [196].

This disease is neurodegenerative, with progressive dementia and rapid onset, with at least two characteristic clinical symptoms: myoclonus, cerebellar or visual signs, pyramidal or extrapyramidal signs, and akinetic mutism, in which post-mortem immunohistochemical detection of PrP^Sc^ is detected [197].

Unfortunately, the involvement of RAS in this type of disease is very limited. Except for possible interactions between oxidative pathogenesis and the evolution of neurotoxicity, it is difficult to make direct connections between the modulation of RAS and prion NDG pathology.

However, bearing in mind that the impact of PRD is quite small in the general economy of NDG diseases, the direct effect of pharmacological modulation of the RAS should be seen through the lens of the actual ”prionoid” theory of neuronal dysproteinemic theory [198].

The interaction with NO synthase, a ubiquitous enzyme related to RAS and involved in vascular metabolism, and nitrinergic nerve mediation, whose function is affected in PRDs, are of relevance to the present discussion [199].

In AD, A-β proteins have the tendency to misfold whenever they come in contact with other A-β variants that have chemical or structural aberrations, similar to when PrP^C^ comes in contact with PrP^Sc^. This type of prion-like behavior seems to be one of the key molecular mechanisms in AD.

In this sequence of events, it has been demonstrated that zinc (Zn) is an essential catalyst of AD protein dimerization. The localization of the Zn atom on the A-β sites is similar with its location on the coupling sites of ACE. This suggested that ACE inhibition, using specific inhibitors such as enalapril, captopril, or lisinopril, may reduce the misfolding effect within A-β proteins. Indeed, targeted inhibition of A dimers using enalapril realized a zinc-dependent oligomerization of these proteins, thus reducing their neurotoxic effects [200].

Given the common pattern of development of prionic proteins, it is possible that ACEIs could be a solution for reducing their neurotoxicity. Further studies using adequate cellular and molecular models may bring new information in this direction.

It is known that metabotropic glutamate receptor (mGluR1 and mGluR5) may form complexes with PrP^C^, and their pharmacologic inhibition may reduce prion neurotoxicity ex vivo and in vivo [130]. Bearing in mind that Ang peptides also act on Ang IV receptors in the brain and have significant effects on memory and behavior, the administration of a pharmacological inhibitor (LAP-4) of metabotropic glutamate receptor may inhibit Ang actions on the acquisition and extinction of behavioral response–effects that may be of use in the study of the behavioral effects of PRD [201].

It is known that increased levels of systemic and cerebral Ang have deleterious effects on cognition [202], and blockage with ACEI or ARB has beneficial effects [203]. Also, essential components of RAS, such as Ang (1–7) and Ang IV, have the ability to inhibit the deleterious effects of RAS hyperactivity on cognitive and behavioral components of brain functioning [204].

Captopril, as an inhibitor of ACE, has a wide range of uses in cardiovascular and other diseases [205]; on the other hand, it is also an inhibitor of the L-Ca^2+^ channel, through the IP3 [173]. Thus, it may be able to inhibit the neurotoxicity of prion-induced misfolding by modulating calcium homeostasis. It has been experimentally shown that captopril administration inhibits PrP^Sc^-induced autophagy in human and murine cell cultures, thus diminishing the apoptotic pathway induced by PrP^C^ misfolding via the AMPK [174]. These beneficial effects have been also identified in relation with the dopaminergic nigro-striatal pathway in PD [206], AD [164], and stroke [207]. Such effects may also be beneficial in prion-induced neurodegeneration (Table 6).

In the last few years, the COVID-19 pandemic prompted a multitude of investigations on the pathogeny of this new virus and also on the patent neurological disturbances shown in a series of cases, the infamous ”long COVID” [208] and its co-involvement with the RAS system [209]. A series of experimental results demonstrated that SARS-CoV-2 penetration is made at the level of the ACE receptor, mediated through the spike protein [210] connecting with the ACE2 receptor in the presence of calcium [211]. The deleterious effects on the CNS are similar to viral encephalitis or systemic inflammation, but some suspect an involvement of protein misfolding with an evolution towards NDG disease, with cognitive deficiencies or motor deficits [212,213].

The chronic use of Ang blockers was incriminated for increasing the risk of infection with the SARS-CoV-2 virus and in the unfavorable evolution of the disease in patients subjected with ACE inhibitors or ARBs for hypertension, cardiac disease, or diabetes, due to the upregulation of the ACE receptor following long-term inhibition. It has been demonstrated that captopril and telmisartan do not significantly alter the membrane expression of ACE2 at the lung or kidney level; however, studies on neuronal ACE are not yet finalized [214].

Several lines of evidence suggest that the neurological aspects of long COVID may point toward degeneration of neural targets [215]. ACE implication may be of interest because SARS-CoV-2 infection can impair the disassembly of host stress granules (SGs) and promote the aggregation of SG-related amyloid proteins, which may lead to an increased risk of neurodegeneration [216].

## 9. Perspectives, If Any

There is a known risk that chronic hyperactivity of RAS will induce an increase in the overall concentration of Ang II throughout the body. This can affect the nervous system and increase the risk of NDG disease. It is still debated whether RAS hyperactivity is a causal factor in the etiopathogeny of NDG disease or merely an aggravating one. On the other hand, the sheer increase in the amount of Ang II could be a source for increased levels of neuroprotective Ang (1–7), Ang IV, or alamandine. The predominance of ARB over ACEI as a neuroprotective therapy suggests that the amount of Ang II present at the CNS level is less important than the receptors on which it couples. This suggests a very important aspect of the neuroprotective effects of AT_2_Rs.

Also, most of the data presented suggest a very important role of the smaller Ang peptides, such as Ang (1–7), Ang IV, and alamandine; however, as substances that specifically increase their production are not yet available, further studies are necessary for ascertaining their efficacy in preventing or treating NDG diseases [217].

A recent meta-analysis that included 15 studies based on data from more than 3 million subjects over a period of more than 5 years concluded that the use of ARBs led to a significant reduction in the risk of dementia. including AD; however, ACEI therapy did not lead to the same results. Future studies should focus on comparisons between therapeutic agents on their ability to cross the BBB [65].

Another meta-analysis that tried to identify variations in the nucleotides of RAS peptides in several NDG diseases was not able to identify direct associations between these and any of the 14 RAS peptides investigated. Vague associations with the PRR were observed in AD, PD, and MS, which led to the conclusion that RAS components were not causative in any of the NDG diseases investigated [218].

From the practical point of view, there are a few topics to be addressed in what concerns RAS modulators and their practical applications. These include:

- BBB penetration depends on molecular weight and lipophilicity; there is a seminal study from 2005 that investigates brain penetration and dosages for several widely-used ACEI, demonstrating that there is only a reduced correlation between lipophilicity and effects on the nervous system, whereas molecular weight seems to be more important [219,220]
- Dosage–nervous effects may need higher amounts than usual, even 10–15 times more, which makes them practically useless due to their vascular effects and side effects [220].
- Several studies have suggested that only about half of the ACE in use today have a good enough BBB penetration to have an effect on neurodegeneration, among which are captopril (most of the studies), trandolapril [220], lisinopril [149], ramipril [221], and perindopril [222]. The rest have not been selected for testing; however, a rigorous testing and classification of those drugs is yet to be made.
- ARB usually have bigger molecules than ACEI [223], suggesting worse passage through the BBB; however, there are several more lipophilic drugs from this family, such as telmisartan, candesartan, and losartan, that seem to readily penetrate and have better effects than ACEI on various parameters of NDG diseases [60,68,83,136]. Until the appearance of metanalyses that can compare these parameters and better pharmacodynamic studies for all pharmacologic modulators of the RAS, only anecdotal evidence is available and patients cannot systematically benefit from these substances.

**Figure 1:**
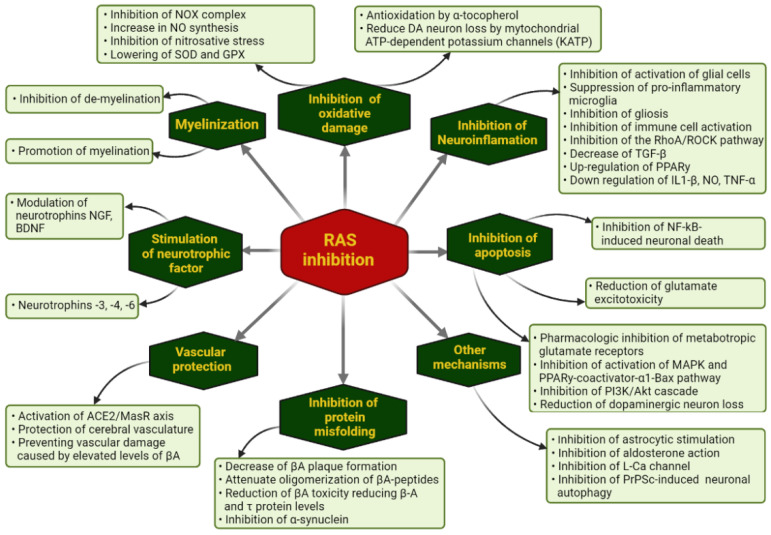
Ways through which pharmacological RAS inhibition may modulate the evolution of NDG diseases.

From the data summarized above, it seems that there is a very important overlap in most NDG diseases from a pathophysiological standpoint. All of them lose neurons in a generalized or localized manner due to the same factors, presented in Figure 1, and all of those pathophysiological mechanisms seem to be affected by the RAS and its modulators.

Gene-set enrichment analysis (GSEA) is a new method of evaluation and drug repurposing, which uses computerized algorithms to search for and identify commonly used drugs that can be repurposed for the treatment of rare and very specific conditions. These substances, often well known, are given a decreasing GSEA score, corresponding to their presumed usefulness in a group of diseases. Conversion enzyme inhibitors have been considered among these potentially useful drugs, with enalapril, fosinopril, quinapril, and moexipril being, in order, the most usable for MND [224,225].

Drug repositioning is among the most modern approaches in pharmacology and therapeutics and several researchers have already used this approach to identify families or individual drugs for the treatment of NDG diseases. The main benefit in such an approach is that this drug or drug family is already in use, and as such, has already-known pharmacokinetics and pharmacodynamics, as well as toxicological characteristics. This approach works from two directions:

- Known mechanism of action that has achieved new dimensions once the research into the etiopathogenesis of NDG disease has sufficiently been advanced (such as prion diseases and rare diseases, such as familial narcolepsy or Friedreich ataxia, and their RAS connections).
- Recognizing new targets from meta-analyses and retargeting studies of known or old drugs. Computerized studies are a great asset because deep-learning arrays and other information technology approaches are becoming increasingly more available [225,226,227,228,229].

An example of repurposing ARB and ACEI use would be in targeting the risk for breast cancer recurrence and management of cardiovascular diseases (CVDs) in postmenopausal women [230,231]. Indeed, ACEIs suppressed vascular endothelial growth factor (VEGF) expression, VEGF-induced angiogenesis, and tumor growth and ARBs also showed similar effects in certain cancer cell lines and animal cancer models [232]. However, subsequent meta-analyses showed no significant association between the use of ARBs and new cancer risk [233].

An exhaustive review presents how ACEIs can be used to treat AD, based on the medical genetics of targets [234]. Another seminal study concludes that the use of ARBs with BBB-penetrating properties during very long time-spans has protective effects on patients with PD and hypertensive or cardiac disease [60]. However, all authors caution that “the complete pharmacological spectrum and therapeutic efficacy of individual ARBs have never been systematically compared, and the neuroprotective efficacy of these compounds has not been rigorously determined in controlled clinical studies” [235]. It is extremely important not to mistake a perspective with certitude, and we are not furthering any hypotheses.

## 10. Conclusions

There is strong evidence that dysregulation of brain RAS has an important role in BNDs. The exacerbation of neuroinflammation, which is considered a key factor in several brain disorders, such as AD, PD, or MS, is explained with AT_1_R activation through Ang II/AT_1_R signaling. Activation of the ACE2/Ang (1–7) neuronal axis/Mas R promotes neuroprotection via antioxidant and anti-inflammatory effects and could have great potential om the development of new therapeutic options for pathologies including PD, AD, MS, HD, MND, or PRD.

There are several papers that have assumed the very difficult task of identifying and enumerating the many RAS modulators that are on the market that may be of use in improving the evolution of NDG diseases. Their protective effects are associated with an increase in vascular protection, inhibition of oxidative damage, neuroinflammation or neuronal apoptosis, stimulation of neurotrophic factors or inhibition of astrocytic hyperstimulation, myelin protection, and inhibition of neuronal protein misfolding.

Most of these studies were unable to draw clear conclusions, due to many reasons, some of which were listed above. There is no common stance of clinicians in this problem, and there is a lack of a unifying theory concerning the pathogenesis of various groups of NDG diseases.

In this paper, we have tried to offer a more unified view of the way through which these drugs could be repurposed to increase the quality of life and survival of NDG patients. The declared scope of this review was to gather all the most recent pieces of experimental evidence concerning the RAS involvement in NDG diseases, in the hope that such quantitative accumulations may lead to a qualitative leap in the development of the therapy or diagnoses of these diseases.

## Figures and Tables

**Table 1:** Protective mechanisms of RAS pharmacological modulators via an increase in vascular protection.

Mechanism of Protection	Biological Pathway	Group of Drugs	Substances	Diseases
Vascular protection	Activation of ACE2/MasR axis [64]	ACEI	Captopril	PD AD
	Protection of cerebral vasculature [53]	ARB	Candesartan and losartan	PD AD
	Prevention of vascular damage caused by elevated levels of A-β [65,66]	ARB/ACEI ARB higher than ACEI	Losartan, olmesartan, and valsartan	AD

**Table 2:** Protective mechanisms of RAS pharmacological modulators via the inhibition of neuroinflammation.

Mechanism of Protection	Biological Pathway		Group of Drugs	Substances	Diseases
Inhibition of neuroinflammation	Inhibition of activation of glial cells [70,71]		ARB	Candesartan	MS AD MND
	Suppression of pro-inflammatory microglia	[72,73,74]	ACEI	Captopril	MS
		[73]	AT_2_R agonist C21	C21	MS
	Inhibition of gliosis	[75]	HGF/cMET receptor or Ang IV/AT_4_R	Ang IV/AT_4_R antagonists Divalinal-Ang IV; norleual-Ang IV	PD AD MND HD
				Ang IV/AT_4_R agonist Nle1-Ang IV [75]	PD AD
	Stimulation of new synapse formation	[75,76]	AT_4_R agonist	ATH-1017	
		[77]	Ang IV/AT_4_R /IRAP	IRAP modulator Dihexa	HD AD
	Inhibition of immune cell activation [78,74]		ACEI	Lisinopril	MS
	Inhibition of the RhoA/ROCK pathway and decrease in TNF-α [67,79]		ACEI	Perindopril	MS
	Decrease in TGF-β [80]		Renin inhibitor	Aliskiren	MS
	Upregulation of PPAR-γ [81]		ARB	Telmisartan	
	Downregulation of IL1-α, NO, and TNF-α [82]				

**Table 3:** Protective mechanisms of RAS pharmacological modulators via the inhibition of oxidative damage.

Mechanism of Protection	Biological Pathway		Group of Drugs	Substances	Diseases
Inhibition of oxidative damage	Inhibition of NOX complex	[89]	ARB	Irbesartan	AD PD MND
		[90]		Telmisartan	AD PD MND
		[89,91]		Olmesartan	PD HD
	Increase in NO synthesis [14,26]		ARB	Losartan	
	Inhibition of nitrosative stress [92]		ACEI	Captopril	MND
	Lowering of SOD and GPX [93]				
	Antioxidation by α-tocopherol [94]			Vit E	ALL
	Reduce DA neuron loss by mytochondrial ATP-dependent potassium channels (K_ATP_) [55]			Azilsartan	PD

**Table 4:** Protective mechanisms of RAS pharmacological modulators via the inhibition of neuronal protein misfolding.

Mechanism of Protection	Biological Pathway	Group of Drugs	Substances	Diseases
Inhibition of protein misfolding	Decrease in A-β plaque formation [83]	ARB	Losartan	AD
	Attenuation of oligomerization of A-β peptides [84]	ARB	Valsartan	AD
	Reduction in A-β toxicity [95]	Renin inhibitor	Aliskiren	AD
	Reduction in A-β and τ protein levels [85]	ARB	Telmisartan	AD
	Inhibition of α-synuclein [98]	ARB	Telmisartan	

**Table 5:** Protective mechanisms of RAS pharmacological modulators via the inhibition of neuronal apoptosis.

Mechanism of Protection	Biological Pathway	Group of Drugs	Substances	Diseases
Inhibition of apoptosis	Inhibition of NF-κB-induced neuronal death [126,127]	ACEI	Perindopril	AD PD HD
	Inhibition of NF-κB-induced neuronal death [126,127] Reduction of glutamate excitotoxicity [128,129]	ACEI	Trandolapril	HD
			Temocapril	ALS MS
	Reduction of glutamate excitotoxicity [128,129] Pharmacologic inhibition of metabotropic glutamate receptors [130]	ACEI	Captopril	HD
			Ramipril	HD
			Perindopril	HD
			LAP-4	PRD
	Inhibition of activation of MAPK and PPARγ-coactivator-α1–Bax pathway [131,132]	ARB	Telmisartan	
	Inhibition of PI3K/Akt cascade [133,134]			
	Reduction of dopaminergic neuron loss [135,136]	ACEI	Captopril	PD
	Reduction of dopaminergic neuron loss [135,136]	ARB	Losartan	PD

**Table 6:** Protective mechanisms of RAS pharmacological modulators via stimulation of neurotrophic factors, inhibition of astrocytic stimulation, or other mechanisms.

Mechanism of Protection	Biological Pathway	Group of Drugs	Substances	Diseases
Stimulation of neurotrophic factors	Modulation of neurotrophins–NGF, BDNF [5,170]	ARB/AT_2_R blockers	Candesartan PD123319	
	Neurotrophins -3, -4, -6 [171,172]			
Inhibition of astrocytic hyperstimulation	Ang II stimulates astrocytes to secrete aldosterone, which is neurotoxic [169]	ARB	Valsartan	HD
	Inhibition of aldosterone action [169]	Aldosterone receptor blocker	Eplerenone	HD
Other mechanisms	Inhibition of L-Ca^2+^ channel [173]	ACEI	Captopril	PRD PD AD Stroke
	Inhibition of PrP^Sc^-induced neuronal autophagy [174]	ACEI	Captopril	PRD PD AD
				Long COVID
Myelin protection	Inhibition of de-myelination [73]	ARB	Candesartan	MS
	Promotion of myelination [125]	ACEI	Captopril	MS

## Data Availability

Not applicable.

## Abbreviations

3-NP	3-nitropropionic acid
6-OHDA	6-hydroxydopamine
Ach	Acetylcholine
ATP	Adenosine triphosphate
AD	Alzheimer’s Disease
APP	Amyloid precursor protein
A-β	β-amyloid
Ang	Angiotensin
ARB	Angiotensin receptor blocker
ACE	Angiotensin-converting enzyme
ACEI	Angiotensin-converting enzyme inhibitor
Angt	Angiotensinogen
ALS	Lateral amyotrophic sclerosis
BBB	Blood-brain barrier
BND	Brain neurodegenerative disease
BK	Bradykinin
PrP^C^	Cell prionic protein
CNS	Central nervous system
CGC	Cerebellar granule cells
CSF	Cerebrospinal fluid
CVD	Cardiovascular diseases
CVO	Circumventricular organ
C21	Compound 21
DAG	Diacylglycerol
DMT	Disease modifying treatment
DA	Dopamine
EAE	Experimental autoimmune encephalomyelitis
GSEA	Gene-set enrichment analysis
GPI	Glycosyl-phosphatidyl-inositol
HGF/c-Met	Hepatic Growth Factor/tyrosine kinase type I receptor
HTT	Huntingtin
HD	Huntington’s Disease
IB	Inclusion bodies
IP_3_	Inositol triphosphate
IRAP	Insulin-regulated aminopeptidase
JG	Juxtaglomerular
LAS	Lateral amyotrophic sclerosis
MPTP	Methyl-phenyl-tetrahydropyridine
K_ATP_	Mitochondrial ATP-dependent potassium channel
MAPK	Mitogen-activated protein kinase
MND	Motor Neuron Disease
MS	Multiple Sclerosis
mHTT	Mutant huntingtin protein
MOG	Myelin oligodendrocyte glycoprotein
NK	Natural killer
NDG	Neurodegenerative
NADPH	Nicotinamide adenine dinucleotide phosphate
NO	Nitric oxide
NOS	Nitric oxide synthase
NOX	Nicotinamide adenine dinucleotide phosphate oxidase
NF-κB	Nuclear factor-κB
PD	Parkinson’s Disease
PPAR-γ	Peroxisome proliferator-activated receptor-γ
PIP_2_	Phosphatidylinositol biphosphate
PRD	Prion Disease
PRR	Prorenin receptor
PKC	Protein kinase C
PLP p139–151	Proteolipid protein 139–151 peptide
ROS	Reactive oxygen species
R	Receptor
RRMS	Relapsing remitting MS
RAS	Renin-angiotensin-aldosterone system
SG	Stress granules
SPN	Spiny projection neurons
SN	Substantia nigra
SMN	Survival motor neuron
TSP-1	Thrombospondin-1
TJ	Tight junction
TGF-β	Transforming growth factor-β
TNF-α	Tumor necrosis factor-α
WT	Wild-type
VEGF	Vascular endothelial growth factor
Zn	Zinc
